# Mapping Surface Charge Distribution of Single-Cell via Charged Nanoparticle

**DOI:** 10.3390/cells10061519

**Published:** 2021-06-16

**Authors:** Leixin Ouyang, Rubia Shaik, Ruiting Xu, Ge Zhang, Jiang Zhe

**Affiliations:** 1 Department of Mechanical Engineering, University of Akron, Akron, OH 44325, USA; lo10@uakron.edu (L.O.); rx7@uakron.edu (R.X.); 2 Department of Biomedical Engineering, University of Akron, Akron, OH 44325, USA; rs169@uakron.edu (R.S.); ge10@uakron.edu (G.Z.)

**Keywords:** surface charge mapping, cell surface charge, charged nanoparticle

## Abstract

Many bio-functions of cells can be regulated by their surface charge characteristics. Mapping surface charge density in a single cell’s surface is vital to advance the understanding of cell behaviors. This article demonstrates a method of cell surface charge mapping via electrostatic cell–nanoparticle (NP) interactions. Fluorescent nanoparticles (NPs) were used as the marker to investigate single cells’ surface charge distribution. The nanoparticles with opposite charges were electrostatically bonded to the cell surface; a stack of fluorescence distribution on a cell’s surface at a series of vertical distances was imaged and analyzed. By establishing a relationship between fluorescent light intensity and number of nanoparticles, cells’ surface charge distribution was quantified from the fluorescence distribution. Two types of cells, human umbilical vein endothelial cells (HUVECs) and HeLa cells, were tested. From the measured surface charge density of a group of single cells, the average zeta potentials of the two types of cells were obtained, which are in good agreement with the standard electrophoretic light scattering measurement. This method can be used for rapid surface charge mapping of single particles or cells, and can advance cell-surface-charge characterization applications in many biomedical fields.

## 1. Introduction

The significance of measuring and visualizing cell surface charge has gradually been recognized during the past decade. Cell surface charge is determined by the composition and dynamic status of the cell membrane. It may differ among species, cell types, benign cells, or differentiation states [1]. Therefore, it could be used as a marker for cell analysis and diagnostics. Recently, one paper has targeted the surface charge characteristics of cancer cells as novel biomarkers for cancer detection and treatments [2]. The variation of cell surface charge has also been reported for various types of stem cells during the differentiation process [3]. It has been widely confirmed that cell surface charge impacts many membrane-regulated cell functions such as endocytosis, muscle cell contraction, nutrient transport (T-cell activation) [4,5], and insulin release [6,7,8]. However, the direct relationship and underlying mechanism between cell surface charge and cell phenotype or caused cell response has been studied less as they require measurement and surface charge mapping in living cells. The measurement and mapping of cell surface charge remains a significant challenge due to the lack of robust techniques capable of micro-and nano-scale measurements in complex environments. The electrophoretic light scattering method remains the dominant method for measuring zeta potential or bulk surface charge of micro or nanoparticles. However, this method usually detects the motions of a group of cells; it is challenging to identify zeta potential for single cells [9], not to mention the surface charge distribution on a cell. Recently Ni et al. developed a microfluidic sensor based on resistive pulse sensing to assess single cells’ surface charge and sizes [10]. However, it still cannot measure the cell surface charge distribution. Atomic force microscopy (AFM) has been used to map living cells’ surface charge [11,12,13,14]. When a charged AFM tip is brought sufficiently close to a target surface (e.g., cell membrane) within the double diffusion layer (DDL) of the target surface, the electrostatic interaction force, arising from the overlap of the double layers, is directly related to the charge density of the two surfaces. However, it is difficult to derive the local surface charge density from the force−distance curves as various forces can act on the probe simultaneously [15]. In addition, although the surface charge dominates the measured force when the AFM tip is positioned within DDL (nanometers from the substrate), such a tiny distance is difficult to control. Hence mapping the surface charge of a cell with AFM tends to be particularly slow and challenging.

Recently, scanning ion conductance microscopy [1,16,17,18] and scanning photo-induced impedance microscopy [18] have been used for quantitative particle and cell surface charge mapping. This method employs a single nanopipette to measure cell topography and surface charge density based on the ion current rectification (ICR) principle [19,20]. However, for each cell’s surface charge measurement, the nanopipette needs to approach the DDL of the cell first, and return to neutral position afterward by trial-and-error; this procedure is labor-intensive and takes prohibitively long to scan the cell surface point by point. Therefore, this technique cannot perform rapid surface charge characterization. In general, there lacks a rapid approach capable of a rapid mapping of the surface charge of living cells, which can discover the unrevealed roles of cell surface charges on cell properties and functions.

**Figure 1:**
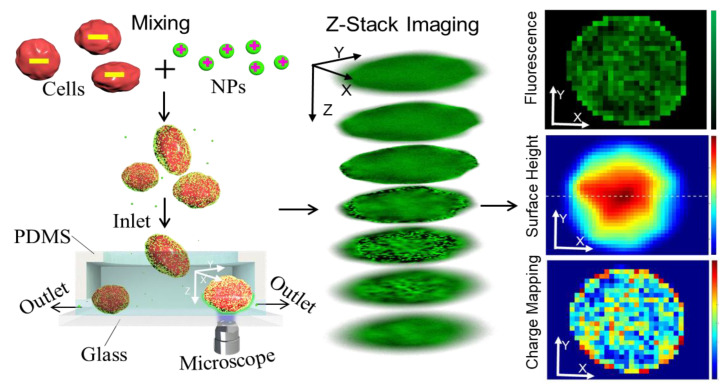
Illustration of the method for a single cell’s surface charge mapping. The cell suspension is mixed with nanoparticles, followed by incubation, washing of unbonded NPs, and capture of cells. The positively charged NPs are attracted onto the wall of the cell due to the electrostatic force. Z-stack images of cells bonded with NPs are captured by a fluorescence microscope, which are processed to map the fluorescence distribution, and thus the surface charge distribution of single cells.

The interaction between nanoparticles and cells has been used for cell diagnosis and imaging [21,22], owing to the stable properties of nanoparticles (e.g., shapes, sizes, and surface charges) [23,24,25,26,27]. This article reports a rapid cell surface charge mapping method based on the interactions of cells and fluorescent nanoparticles as the methods shown in Figure 1. Since cells usually have a negative surface charge, due to the phospholipid bilayer composition [28,29], and positively charged fluorescent nanoparticles were chosen to bond the fluorescence to the cell surfaces. By measuring the fluorescence distribution on the cell surface, surface charge mapping of a single cell can be obtained. Results showed that this method could rapidly map the cells’ surface charge at a low cost.

## 2. Materials and Methods

### 2.1. Detection Principle

The rapid cell-surface-charge mapping method is based on the interactions between cells and positively charged nanoparticles. Positively charged fluorescent nanoparticles are bonded to the negatively charged cells. The higher the local surface charge on the cell membrane, the larger the number of bonded nanoparticles locally, and the higher the local fluorescence intensity. Hence the single cell’s surface charge mapping can be obtained by measuring the fluorescence intensity’s distribution on the cell surface. After mixing the NPs with cells, the single cell’s surface charge mapping procedure is as follows: first, we take a series of Z-stack fluorescent images of a cell with a fluorescence microscope. Second, we analyze the Z-stack fluorescent images and determine the fluorescence intensity distribution on the cell surface. Finally, after calibrating the quantified relationship between the fluorescence intensity and the surface charge density, a single cell’s surface charge mapping is obtained. We selected the 200 nm NPs (0.2 μm amine-modified microspheres, manufactured by FluoSpheres, purchased from Fisher Scientific, Waltham, MA, USA) with positive surface charge. The NPs, made of polystyrene, are covered by amine function groups that generate a positive surface charge.

To record the cell’s surface profile and the fluorescent intensity of the cell surface bonded with NPs, we imaged the same cell at different focal positions (along the *Z*-axis) to obtain a three-dimensional image in the form of a two-dimensional image stack, based on the maximum intensity projection [30,31]. A series of fluorescent photos were obtained by multi-layer photography in the *Z*-axis direction with a small step size of 1 μm. These Z-stack images were combined to determine the cell surface morphology and the fluorescence intensity, following the procedure described in other articles [30,31]. The surface profile involved selecting the maximum intensity position along the *Z*-axis for each X, Y position. The surface index or height was recorded as an index map to obtain the cell surface profile and calculate the surface area size. The image composed of fluorescence intensity values corresponding to the surface height map is also gained. Therefore, the surface charge mapping of single cells was obtained from the fluorescent images adapting the correlation between fluorescence intensity and charge density.

### 2.2. Calibration of the Relation between Fluorescence Intensity and Surface Charge Density

According to the Gouy−Chapman theory [32], zeta potential represents the cell and particle’s surface charge density in a solution. The relation between the net charge density and the zeta potential can be described by the Gouy−Chapman equation [9,15,32,33,34,35]:

(1) $$\sigma_{charge}=\sqrt{8cN_{A}\varepsilon_{r}\varepsilon_{0}\kappa_{B}T}\;\sin h\left(\frac{e\xi }{2\kappa_{B}T}\right)$$

where *σ_charge_* is the surface charge density, *c* is the ion concentration, *N_A_* is the Avogadro constant, *ε_r_* is the relative dielectric permittivity of the solution, *ε*_0_ is the vacuum permittivity, *κ_B_* is the Boltzmann constant, e is the charge on a proton, *ξ* is zeta potential, and *T* is the absolute temperature.

**Figure 2:**
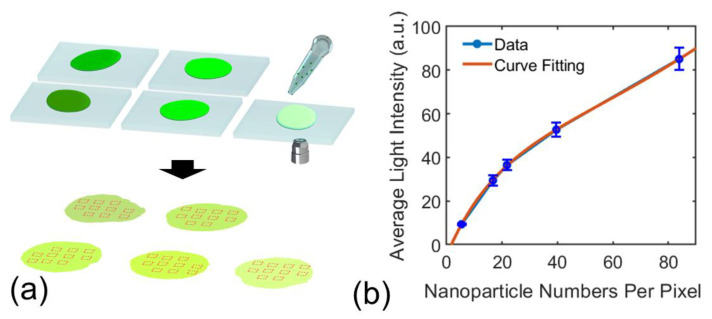
(**a**) Illustration of calibrating the relation between the nanoparticles’ fluorescence intensity and nanoparticle numbers. (**b**) The relation between the measured average fluorescent light intensity of nanoparticles and the number of nanoparticles per unit area (1 µm × 1 µm).

As more NP attachment on a local area leads to a higher fluorescence intensity [36], the fluorescence intensity at this area can represent the number of attached NPs. Next, we obtained the relation between the fluorescence intensity per unit area and the number of NPs by measuring the fluorescence intensity of a set of NP droplets with different NPs concentrations. As shown in Figure 2a, NP droplets (each with a volume of 3.2 μL) were pipetted onto a clean glass cover slide separately. After the droplets were nearly dry, we took pictures of these droplets to measure their area size and randomly selected ten areas within each droplet to measure each droplet’s fluorescence intensity per unit area. Note that these droplets’ shapes could be irregular; we calibrated the images’ pixel size in micrometers. By counting the pixel number of each NPs’ dried droplet, we could obtain the droplet’s area. The number of NPs per pixel were calculated by the following equations:

(2) $$N_{NP}=\frac{1}{A_{d}}\frac{6C\cdot 10^{12}}{\rho \pi \phi^{3}}V$$

where *N_NP_* is the number of nanoparticles per pixel (1 µm × 1 µm), *A_d_* is the area of the dried droplet, *C* is the concentration of suspended nanoparticle in g/mL, *ϕ* is diameter of nanoparticles in micrometer, *ρ* is the density of nanoparticles in g/mL (1.055 g/mL), and *V* is the droplet volume (3.2 µL).

From Equation (2) we can calculate the number of NPs per pixel in each NP droplet. Thus, the relationship between the fluorescence intensity per unit area and the number of NPs per unit area was established (see Figure 2b). The results show that the fluorescence intensity increased approximately exponentially with the increase in the number of NPs; a fitting curve was obtained to gain NPs number from fluorescence intensity, as

(3) $$N_{NP}=0.0878\cdot I^{1.5438}\;$$

where *N_NP_* is the number of NPs per pixel (1 µm × 1 µm), and *I* is fluorescence intensity per pixel.

The average surface charge density of each NP (*σ_NP_*) can be calculated from Equation (1) using its zeta potential. The average zeta potential of NPs in saline was measured to be +1.92 mV from Zetasizer (Nano Z, Malvern Panalytical, Malvern, England, UK). Subsequently, we multiplied the measured σ_NP_ with the NPs’ spherical surface area to gain each NP’s total charge. The charge of each particle is calculated by:

(4) $$q_{NP}=\sigma_{NP}A_{NP}$$

where *q_NP_* is the charge of each nanoparticle, *σ_NP_* is the charge density of NPs, and *A_NP_* is the surface area of each nanoparticle.

Using *N_NP_* (converted from the measured light intensity by Equation (3)) and *q_NP_*, we can calculate the cell charge density. We noted that the cell surface is not flat, so the actual surface area of each pixel on the cell surface needed to be calculated from the three-dimensional profile. The cell surface charge density per pixel can be calculated from:

(5) $$\sigma_{\;pixel}=-\frac{N_{NP}\cdot q_{NP}}{A_{pixel}}$$

where *σ _pixel_* is the surface charge density of the pixel in the cell surface, *N_NP_* is number of NPs converted from the fluorescence intensity, *q_NP_* is the charge density of each NP, and *A_pixel_* is the actual area size, which is calculated from the three-dimensional profile.

From Figure 2b, an exponential correlation curve was obtained, which served as the calibration curve to convert the measured light intensity to the number of NPs attached to cell’s surface. We also tracked and measured the droplet’s fluorescence intensity from wet to dry, i.e., opening the excitation laser shielding every 1 min to take the droplet fluorescence image for a 60-min duration. The fluorescence intensity remained nearly unchanged, indicating the dry state’s relationship measure can represent the relationship at the wet state.

### 2.3. Testing Setup and Signal Processing

Cells are collected and resuspended in saline solutions. The 9% (weighted) sodium chloride saline was chosen as the medium, as proteins in the cell culture medium are also negatively charged and would deteriorate the nanoparticles’ ability to attach to the cell membrane. Details on the protocol of cell culture can be found in the cell culture section. The cell concentration was set to be 10^5^ cells/mL, and the NPs concentration was set to be 200 µg/mL. As shown in Figure 1, positively charged particles were added to the cell suspension with gentle agitation. The temperature was kept as ice temperature (0 °C) to prevent the cells from uptaking NPs [28], and to ensure electrostatic interactions between the cell membrane and positively charged NPs were dominant [29]. After adding NPs, 10 min was set for the incubation of NPs and cells. A droplet of 50 µL volume of the mixture was taken into 1 mL saline solution for each type of cells to dilute the unbonded NPs. Next, a PDMS channel was made to separate the cells from the unbonded NPs. The PDMS channel consisted of one inlet reservoir, one connecting channel with a space height of ~3 um, and two outlets (see Figure 1). Once pumped into the filter device, the cells were captured at the bottom of the inlet reservoir: the lower height of the connecting channel prevented the cells from moving to the outlet, while the unbonded NPs with the sub-micrometer size were guided to the outlet. After the separation, the cells were positioned on the inlet reservoir’s bottom (at the edge between the inlet reservoir and connecting channel).

A fluorescence microscope imaged every single cell from the back of the glass substrate. We took 100 Z-stack images of each captured cell at various focal depths (Z positions) with an Olympus Fluorescence Microscope (Model IX71, Olympus, Shinjuku, Tokyo, Japan) equipped with a dichroic fluorescence filter and a standard 100 W Mercury Illuminator. Z-stack images were taken with a step size of 1 μm. The illuminator was calibrated each time and pre-warmed for 20 min. A total of 20× objective lenses and 1.7× eyepieces were used. An AmScope Microscope Camera (AmScope, Irvine, CA, USA) using 50 ms exposure time was connected to the fluorescence microscope to record the Z-stack images. These images were then processed using the Matlab (R2020b) program to generate the fluorescence mapping with a resolution (or pixel size) of 1 μm × 1 μm. From the continuous 100 images, we set the Z value of the fluorescent image with the minimum contour as the zero position (Z0). The contour was gained using a fluorescence intensity threshold, half the maximum fluorescence intensity of each image. For each X-Y position (horizontal panel), we tracked the Z value with maximum fluorescence intensity from the 100 Z-stacks images (vertical positions), from which the Z-index mapping (surface height equals to Z minus Z0) can be obtained for each cell. As a result, we could get the Z value with the maximum fluorescence intensity for every X-Y position to form the X-Y-Z points cloud. For example, on a horizontal position X = 45 and Y = 55, among 100 Z values (Z = 1~100), if the maximum fluorescence intensity is obtained at Z = 50, it indicates this Z coordinate represents the actual height at this X-Y position, as the images at other Z positions are all defocused. This process was repeated for each X-Y position to gain the X-Y-Z coordinates of the cell surface. We then built the cell surface morphology, which was based on the triangulated mesh generated by the given set of X-Y-Z coordinates obtained from the Z-index Mapping. We also recorded the maximum fluorescence intensity for each X-Y position, which was used to create the 2-D fluorescence intensity mapping on a projection plane, and stored it on the projected image. The cell surface area and fluorescence distribution can be calculated from the Z-stack-based three-dimensional profile. Thus, each pixel’s fluorescence intensity was then transferred to the surface charge mapping from the Equations (3)–(5) and the calibration curve shown in Figure 2b. Note that there was background fluorescence in each Z slice image. In the signal processing, the background noise was analyzed and removed to reflect fluorescence by the attached NPs.

Zeta potential measurements for NPs and cells were also conducted to prove the measurement validity of our method. Zetasizer (Nano Z, Malvern Panalytical, Malvern, England, UK) was used for this purpose. Before the measurements, potential transfer standard particles (DTS1235, Malvern Panalytical, UK) were measured to calibrate the instrument. The measured value, −41 ± 4.0 mV, matched well with the given zeta potentials (−42 ± 4.2 mV). The monomodal model was chosen for zeta potential measurements of cells and NPs in saline solution due to the high conductivity. There were at least ten runs to obtain the mean zeta potential value of the target particles.

### 2.4. Cell Culture

Human umbilical vein endothelial cells (HUVECs, Cat. NO: C2519A, Lonza) were cultured using Endothelial cell growth medium-2 Bulletkit^TM^ (EGMTM-2, Lonza, Basel, Switzerland) containing basal medium and the required supplements. Whereas human negroid cervix epitheloid carcinoma cells (HeLa, Cat. NO: 93021013, SigmaAldrich, St. Louis, MO, USA) were cultured in minimum essential medium eagle, with ear (EMEM, SigmaAldrich) supplemented with 2 mM L-glutamine solution Bioxtra (SigmaAldrich), 1% MEM Non-essential amino acid (NEAA, SigmaAldrich), and 10% Fetal bovine serum (FBS, SigmaAldrich). Additionally, 1% penicillin-streptomycin (Fisher Scientific, Waltham, MA, USA) was added to both media. Briefly, the cells were seeded in T75 flasks containing 15 mL of the complete medium at a seeding density of 2500 cells/cm^2^ for HUVECs and 3500 cells/cm^2^ for HeLa, and incubated at 37 °C. After seeding, the media was changed after 16–24 h and then every other day (48 h) until the flasks were about 80% confluent. Once the flasks reached desired confluency, the HUVECs were harvested by using the Reagentpack^TM^ subculture reagents (Lonza) containing HEPES buffered saline solution, trypsin/EDTA 0.025% solution, and trypsin neutralizing solution (TNS). The spent medium was aspirated and washed once with 15 mL of HEPES buffer saline, then 6 mL of trypsin/EDTA was added and incubated at 37 °C for 3 to 5 min. After the cells were detached, 12 mL of TNS was added to the flask, and the cells were pelleted using an Eppendorf centrifuge at 4 °C set at a speed of 200 g for 5 min. HeLa cells were washed once with Dulbecco’s Phosphate-Buffered Salt Solution 1× (DPBS, Fisher Scientific, Waltham, MA, USA), and then 4 mL of 0.25% Trypsin-EDTA solution (SigmaAldrich) was added and incubated at 37 °C for 5 min. After trypsinization, 8 mL of complete EMEM medium was added, and the HeLa cells were pelleted in the centrifuge at 4 °C set at 220 g for 5 min. The cells were counted via staining with Trypan blue solution 0.4% (Fisher Scientific, Waltham, MA, USA), and then resuspended in saline solution (Fisher Scientific, Waltham, MA, USA) to a final working concentration of 105 cells/mL followed by mapping surface charge distribution.

### 2.5. Statistical Analysis

All data points reported as means or error bars (standard deviations) were obtained with 5–60 independent samples. The statistical analysis was carried out using MATLAB (R2020b). Analysis of variance (ANOVA) and Tukey’s post hoc test were used to compare the differences among cell groups. A *p*-value of less than 0.05 was considered to be statistically significant.

## 3. Results

### 3.1. Validation of the Method via Charged Microparticles

**Figure 3:**
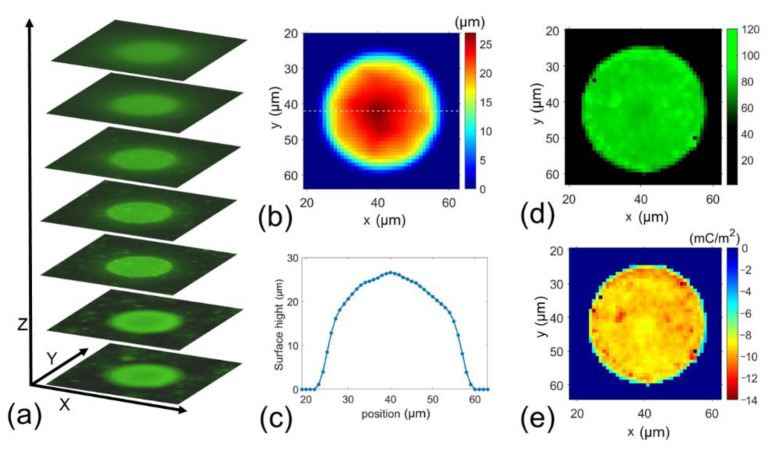
(**a**–**e**) Typical measurement results of surface charge mapping of a 31 µm MP. (**a**) Z-stack images, (**b**) surface height mapping, (**c**) the surface height profile at the centerline located in the MP center in X-direction, (**d**) fluorescent intensity distribution, and (**e**) the surface charge mapping.

First, the 31 μm standard black polyethylene microspheres (MPs, black paramagnetic polyethylene microspheres, manufactured by Cospheric LLC, purchased from Fisher Scientific, Waltham, MA, USA) were used for validating our method. The MPs were diluted in 0.9% saline solution. The particle zeta potentials were measured to be −6.43 ± 0.65 mV in saline solution at a temperature of 0 °C. Fluorescence NPs were then added to the MP suspension to induce MPs-NPs binding via electrostatic attraction. All procedures were operated at 0 °C. We utilized the same procedures for cells as described in Section 2.3. Testing setup and signal processing. Twenty microparticles were measured. A set of 100 Z-stack images were taken and processed for each microparticle. Figure 3a shows typical Z-stack images taken at an MP. Figure 3b gives the surface height index mapping obtained from Z-stack images by projecting its surface height. From Figure 3b, we can obtain the surface height (as shown in Figure 3c) of the centerline located at the particle center at X direction at Figure 3b, and Figure 3d shows the fluorescence intensity distribution. The surface charge mapping (Figure 3e) can be obtained from the calibration curve (see Figure 2b) and Equations (3)–(5).

To validate the measurement, we converted the surface charge mapping to the average charge density for each MP. After NPs are bonded to a microparticle or a cell’s surface, the surface charge is neutralized in any local area. The larger the local surface charge, the more significant the number of bonded NPs. Hence, the average charge density can be measured by measuring the bonded NPs:

(6) $$\bar{\sigma_{cell}}=-\frac{n_{NP}\;\sigma_{NP}\;A_{NP}}{A_{cell}}$$

where $\bar{\sigma_{cell}}$ is the average cell surface charge density, *n_NP_* is the number of nanoparticles bonded to the cell surface, *σ_NP_* is the nanoparticles surface charge density calculated from zeta potential, *A_NP_* is the surface area of a nanoparticle, and *A_cell_* is the surface area of a cell.

**Figure 4:**
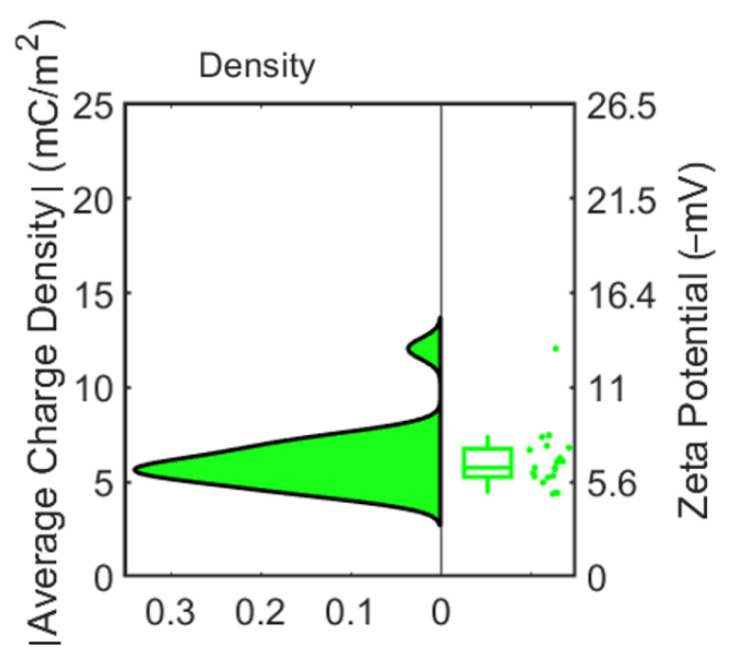
Left: distribution of average charge density of each MP obtained from fluorescence method. Right: boxplot of zeta potential converted from average charge density of MPs.

The average cell surface charge density of each MP can then be obtained. Next, we converted the average charge density to the MPs’ zeta potential using Equation (1). Figure 4 shows the zeta potentials calculated from the mapping results for 20 MPs. The average zeta potential was −6.12 ± 0.90 mV, which is in good agreement with the electrophoretic light scattering measurement by the Zetasizer (Nano Z, Malvern Panalytical, Malvern, England, UK), −6.43 ± 0.65 mV. The slight difference might be a measuring error due to local electric field inhomogeneities [37] and electrophoretic mobility measurement uncertainty from adsorbed macro-molecules [38]. This test validated the accuracy of our method for surface charge mapping of spherical particles.

### 3.2. Single-Cell Surface Charge Mapping

**Figure 5:**
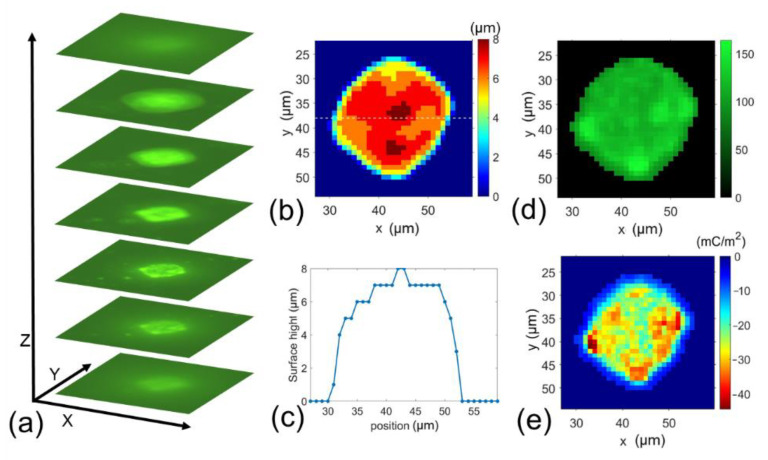
Typical measurement results of surface charge mapping of a Hela cell. (**a**) Z-stack images, (**b**) surface height mapping, (**c**) the surface height profile at the centerline of the cell along X-direction, (**d**) fluorescent intensity distribution, and (**e**) cell surface charge mapping.

**Figure 6:**
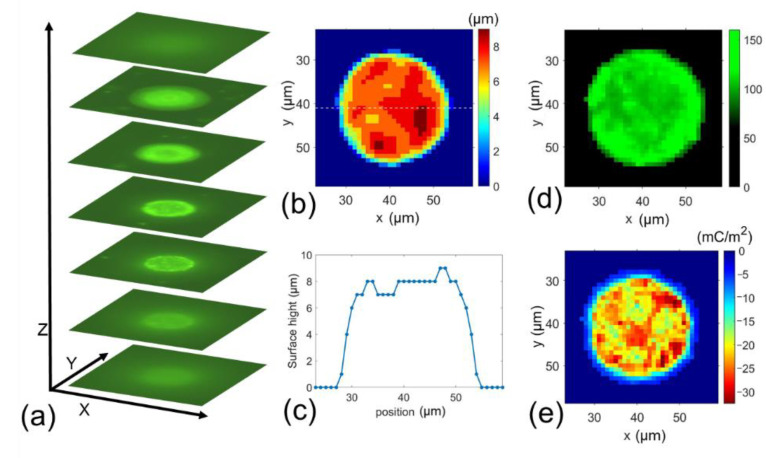
Typical measurement results of surface charge mapping of a HUVEC cell. (**a**) Z-stack images, (**b**) surface height mapping, (**c**) the surface height profile at the centerline of the cell along X-direction, (**d**) fluorescent intensity distribution, and (**e**) cell surface charge mapping.

Next, we used the same procedure to perform the surface charge mapping of two cells (HUVEC cells and Hela cells). HUVECs are human umbilical vein endothelial cells for investigating various normal processes and diseases’ cellular and biochemical behavior. Hela cells are derived from cervical cancer cells and are widely researched as cancer cells’ functions and properties. Once NPs were bonded to the cells, a droplet of this suspension of cells and NPs mixture was pipetted into the saline solution to wash out unbonded NPs, and the device captured cells. A total of 60 HUVEC cells and 60 Hela cells were randomly selected from the cell suspension for measurement. For each cell, we took a set of 100 Z-stack images. Each cell Z-stack image was processed using the procedures described in Section 2.3. Testing setup and signal processing to map the surface charge distribution. Typical results of Hela cells and HUVEC cells are shown in Figure 5a–e and Figure 6a–e, respectively. Both plots showed the method could measure the surface charge mapping and cell topography simultaneously. The surface charge mapping seems HUVEC cells have a more uniform surface charge distribution than Hela.

The surface charge density of a group of cells can be from the zeta potential measurement; our results show that some cells displayed a charge density differing from the average values. This hints that cell surface charge can vary from each cell. More charged particles were bound to cells on some areas of their membrane, indicating the presence of highly negatively charged areas on the cell surface. Figure 5 and Figure 6 display sample images of positively charge particles bound to Hela and HUVEC cells, respectively. Although these cells’ overall surface charge is of a single value, the fluorescent light distribution is not uniform, allowing us to find the higher or lower charged area on the cell surface.

**Figure 7:**
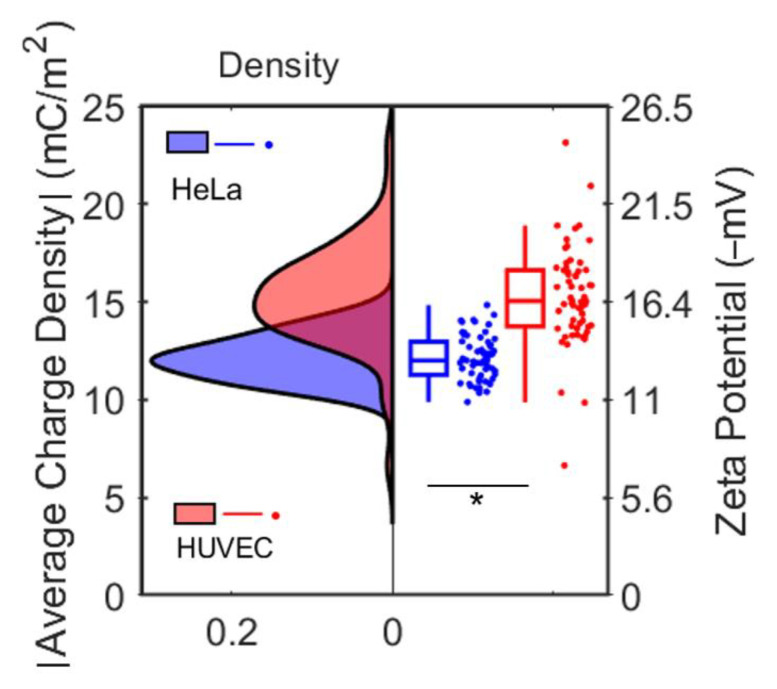
Comparison of the measured average charge density for each cell type, obtained from fluorescence method, and the corresponding zeta potential of HUVEC and Hela cells. Left: distribution of average charge density of cells. Right: boxplot of zeta potential converted from the average charge density of cells. The statistical test showed statistically significant surface charge density difference between Hela cells and HUVEC cells. * *p* < 0.05 (*n* = 60).

Additionally, we conducted zeta potential measurement for both cells using NanoZ. The measured zeta potentials for these two cells were −13.8 ± 1.4 mV and −17.1 ± 1.7 mV. We calculated the average charge density of each Hela and HUVEC cell from the measured fluorescence intensity (see Figure 7), which were −12.14 ± 1.14 mC/m^2^ and −14.9 ± 2.4 mC/m^2^, respectively. Subsequently, they were converted to the zeta potentials using Equation (1), as shown in Figure 7. The average calculated zeta potentials of Hela and HUVEC cells were −13.2 ± 1.2 mV and −16.4 ± 2.6 mV, respectively, which were significantly different and match the NanoZ measurement reasonably well. We conducted a statistical analysis (ANOVA and Tukey’s post hoc test) to compare the surface charge density between HUVEC and Hela cells. The test showed statistically significant surface charge density difference between Hela cells and HUVEC cells with a *p*-value less than 0.05.

**Figure 8:**
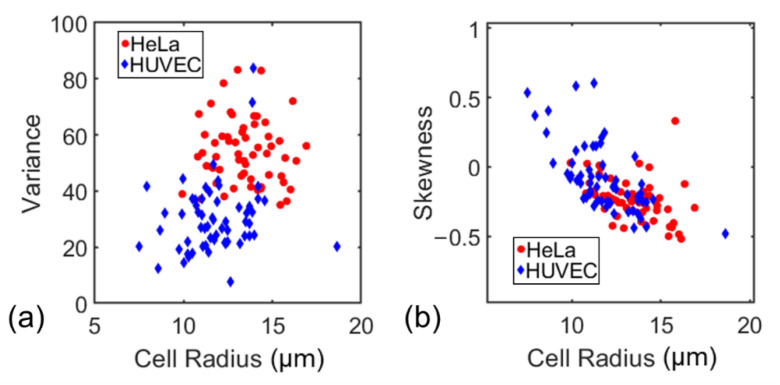
(**a**) The variance of the surface charge density distribution of Hela and HUVEC cells, and (**b**) the skewness of surface charge density distribution of Hela and HUVEC cells.

Next, from the measured surface mapping, we conducted additional statistical analysis for each cell. The variance and skewness of the surface charge density measured on all 1 µm × 1 µm pixels of each cell were calculated (see Figure 8a,b). Variance is a measure of the degree of variation of the data. If the fluorescence intensity value or charge density of each cell surface pixel is close to the average value of the entire surface’s fluorescence intensity, the variance would be slight (i.e., close to zero). The greater the variance of the surface charge density distribution on a single cell’s surface, the more the surface charge is nonuniform. Skewness is a measure of asymmetry around the cell’s mean surface charge density. Negative skewness indicates over 50% of pixels have a less than average fluorescence intensity on the whole cell surface and vice versa. In other words, negative skewness reflects that the average surface charge density is less than the median surface charge density on the cell surface and vice versa.

Figure 8a shows the variance of the surface charge density. HUVEC and Hela’s mean-variance is 54 and 30, respectively, indicating that HUVEC cells have a more uniform surface charge distribution than Hela cells. Figure 8b shows the skewness of all cells that had been measured. It seems most of Hela cells’ skewness is negative with a mean value of −0.23, indicating more pixels have a lower-than-average surface charge density (i.e., the charge is more concentrated in a few areas on the cell surface). Similarly, as half of HUVEC cells’ skewness is positive, with a mean value of −0.05, more surface areas of HUVEC cells have a larger than average surface charge density. As the two types of cells show the difference in the surface charge distribution (e.g., variance, skewness), the characteristic can be used to differentiate the two types of cells.

## 4. Discussion

This method provides quick and accurate single-cell surface chance mapping. This simple method can be integrated on a chip. Compared to surface charge mapping using AFM, the AFM tip must be placed within the double layer of the surface membrane, which is very difficult to control. Additionally, it is challenging to measure the local surface charge density from the force−distance curves accurately due to other forces (e.g., van der Waals interaction force and short-range hydration force [12]). While scanning ion conductance microscopy is another method that can quantitatively map the cell surface charge, this method also employs a single nanopipette that needs to be placed at a working distance as of thirty nanometers from the cell surface [19]—scanning the entire cell surface with such a nanopipette is labor-intensive and time-consuming. While our method provides a rapid single cell surface charge and topography mapping, it does not need expensive equipment, and can be operated with a typical fluorescence microscope in a lab. Note that the variations in the size and zeta potential of NPs will certainly cause measurement uncertainty in the cell surface charge measurement. To reduce the measurement uncertainty, one solution is to use customized nanoparticles with uniform size and zeta potential. It should be noted that charged nanoparticles have been used to quantify surface charge towards cell type detection and cytological analysis [28]. However, these applications mainly measure the bulk surface charge of cells. Our method can provide quantitative measurement for both bulk surface charge and 3D surface charge distribution. The results demonstrate that, in addition to the difference in bulk surface charge, HUVEC and Hela cells have distinct surface charge distribution, suggesting the potentials of using cell surface charge distribution as a new criterion for cell type identification malignant cell detection. It is worth mentioning that using confocal or super-resolution microscopy can certainly reduce the background noise and improve the resolution of Z-stack images, which will ultimately result in higher accuracy of the calibration and the measurement. Nonetheless, our method can still provide decent accuracy and resolution using a common fluorescence microscope available in most labs. While we used NPs with relatively low surface charge density and high concentration in our experiments, we noticed the nonlinear relationship between the light intensity and number of nanoparticles. In our future work, we plan to use NPs with high charge density and low concentration to enable the monolayer or sub monolayer NP attachment on the cell surface; this would lead to a linear relationship between light intensity and local surface charge density. The formation of NP monolayer will also avoid the screening effect possibly caused by multilayer NPs. Cell surface charge reflects the cell membrane’s dynamic status that affects many cell functions such as cell division, signaling, nutrient transport, and movement. Cell surface charge mapping generated by this approach could reveal the charge density in a specific region of the cell membrane, which would significantly advance our mechanistic understandings of surface charge-regulated cell membrane actions. In addition, the method can be used as a novel platform to facilitate targeted drug delivery or cell membrane manipulation using charged nanoparticles in which drugs or biomolecules are encapsulated.

## 5. Conclusions

In conclusion, we demonstrate a new method to map single cells’ surface charge distribution. This method utilizes the electrostatic interactions between positively charged fluorescent nanoparticles and cells. The fluorescence intensity distribution on a single cell’s surface is measured via a typical fluorescence microscope, and converted to cell surface charge mapping. Surface charge mappings of microparticles, Hela cells, and HUVEC cells were measured. Zeta potentials of these particles were back-calculated from the surface charge mapping, which are in good agreement with the commonly used electrophoretic light scattering, proving the new method’s validity. The measure shows that different cell types (e.g., Hela cells and HUVEC cells) have a distinct difference in surface charge distribution in terms of variance and skewness. Compared to other methods, including atomic force microscopy, and scanning ion conductance microscopy, this method leads to quick and accurate single-cell surface charge mapping without expensive equipment and cell modification. With its ability to quickly map a single cell’s surface charge distribution, this method can help reveal the influence of cell surface charge characteristics on cell functions and behavior. It can be potentially used for many applications, including cellular analysis, diagnosis, manipulation, and nanomedicine.

## Data Availability

Available upon reasonable request.
